# Lower expression level of IL-33 is associated with poor prognosis of pulmonary adenocarcinoma

**DOI:** 10.1371/journal.pone.0193428

**Published:** 2018-03-02

**Authors:** Min Yang, Yuehua Feng, Cuihua Yue, Bin Xu, Lujun Chen, Jingting Jiang, Binfeng Lu, Yibei Zhu

**Affiliations:** 1 Department of Immunology, School of Biology and Basic Medical Sciences, Soochow University, Suzhou, China; 2 Department of Immunology, School of Medicine, University of Pittsburgh, Pittsburgh, Pennsylvania, United States of America; 3 Department of Tumor Biological Treatment, The Third Affiliated Hospital of Soochow University, Changzhou, China; 4 Comprehensive Laboratory, The Third Affiliated Hospital of Soochow University, Changzhou, China; 5 Institute of Cell Therapy, Soochow University, Suzhou, China; 6 Jiangsu Key Laboratory of Clinical Immunology, Soochow University, Suzhou, China; University of South Alabama Mitchell Cancer Institute, UNITED STATES

## Abstract

**Objective:**

Lung cancer is one of the deadliest malignancies. The immune checkpoint-blockade (ICB) tumor therapy has led to striking improvement of long-term survival for some lung cancer patients. However, the response rate of immunotherapy is still low for lung cancer. Studying the tumor microenvironment (TME) should shed light on improvement of immunotherapy of lung cancer. Interleukin-33 (IL-33), an “alarmin” cytokine, has been implicated in tumor associated immune responses and inflammatory diseases of the lung. The role of IL-33 in lung cancer progression, however, remains elusive. This study is designed to characterize IL-33 expression in lung tumor tissues and establish the clinical significance of IL-33 in non-small cell lung cancer lung cancer (NSCLC).

**Materials and methods:**

Tumor tissue specimens from patients suffering from NSCLC were analyzed for expression of IL-33 protein by immunohistochemistry and expression of IL-33 and ST2 mRNA by RT-quantitative PCR (RT-QPCR). The expression data were analyzed for their association with clinical and pathological parameters of NSCLC. In addition, the association between expression levels of IL-33 mRNA and patient survival was determined using 5 independent expression profiling datasets of human lung cancer.

**Results and conclusion:**

The expression levels of IL-33 and ST2 were significantly down-regulated in both adenocarcinoma and squamous cell carcinoma of the lung when compared to adjacent normal lung tissues. In addition, the level of IL-33 protein was inversely correlated with tumor grade and size. Moreover, analysis of TCGA and GEO lung cancer expression datasets revealed that higher expression levels of IL-33 mRNA were correlated with longer overall survival of patients suffering from adenocarcinoma of the lung. These data indicate that the expression levels of IL-33 are inversely associated with lung cancer progression, consistent with the hypothesis that IL-33 is involved in immune surveillance of NSCLC.

## Introduction

Cancer progression is inhibited by tumor immune surveillance, because cancer cells express unique tumor antigens, which trigger T cell-mediated antitumor immune responses [1–5]. In order to prevent T cell recognition, tumor establishes immune tolerance or ignorance of tumor antigens through multitudes of mechanisms such as insufficient tumor antigen processing, downregulation of MHC molecules, and decreases of co-stimulatory molecules and cytokines. In addition, tumor cells suppress active antitumor immune responses through numerous means such as down-regulation of antigen presentation and immune stimulatory molecules, up-regulation of immune suppressive cytokines and “checkpoint” molecules, and nutrient deprivation [6]. As a result, the immune system cannot mount effective immunity against tumor cells in cancer patients. Overcoming immune tolerance and suppression is critical for the success of immunotherapy of cancer.

Among the immune stimulatory molecules, epithelial cell-derived cytokines play an important role in initiating and sustaining antitumor immunity [7]. Interleukin-33 (IL-33), an “alarmin” and a member of the IL-1 family of cytokines, plays important roles in multiple physiological and pathological conditions. IL-33 is constitutively expressed in the nuclei of tissue lining cells, mainly epithelial and endothelial cells, and functions as a damage-associated pattern molecule (DAMP) to mediate tissue immune responses [8]. IL-33 has been shown to exert strong antitumor activities via type 1 lymphocytes such as CD8^+^ T cells, Th1 cells, NK cells, and γδT cells [9–11]. However, IL-33 can also promote tumorigenesis through myeloid derived suppressor cells [12]. The exact role of IL-33 during human epithelial tumor progression is not well understood.

Lung cancer is one of the deadliest malignancies in the world and approximately 85% are NSCLC [13]. Despite the impressive clinical efficacy of the ICB immunotherapy for some patients, majority of lung cancer patients have yet benefited. Understanding the immune characteristics of lung tumor tissues should help designing better immunotherapeutic approaches. Since IL-33 has been shown to be involved in various lung diseases, we set out to study IL-33 expression during human lung cancer development. To this end, we used immunohistochemistry and RT-QPCR to establish the nature of IL-33 expression in NSCLC. We then determined the association between expression levels of IL-33 and clinical and pathological parameters of NSCLC.

## Materials and methods

### Patients and tissue samples

A total of 127 lung cancer tissue specimens and adjacent normal tissues were obtained from patients who received surgery for lung cancer at the Department of Cardiothoracic Surgery of the Third Affiliated Hospital of Soochow University from Jan 2014 to Feb 2015. One part of each tissue was snap-frozen immediately in liquid nitrogen after resection, and the other part of the tissue was fixed in 10% (v/v) formalin and embedded in paraffin for immunohistochemical investigation. All of the lung cancer tissues were histologically identified as non-small cell lung cancer, including adenocarcinoma (80 cases) and squamous cell carcinoma (47cases), evaluated by senior pathologists. The protocol for the present study was approved by the ethics committees of the Third Affiliated Hospital of Soochow University hospital.

### Total RNA isolation and RT-qPCR

Total RNA was extracted using the guanidiniumthiocyanate method [14] from both NSCLC tumor tissues and their adjacent normal tissues. The quality of RNA was evaluated by the agarose gel electrophoresis and absorbance measurement at 260/280nm. First-strand cDNA was synthesized from total RNA using the first strand cDNA synthesis kit (Fermantas). Real-time PCR was performed with SYBR green (Applied Biosystems). Cycling conditions were set as following: 30 sec at 95°C, followed by 40 cycles of 95°C for 5 sec and 60 °C for 20 sec, after that, a melting program at 95°C for 0 sec, 65°C for 15 sec and increased to 95°C at a temperature transition rate of 0.1°C /s was performed to monitor the amplification specificity. Samples were amplified simultaneously in triplicates in one-assay run. Analysis was performed by sequence detection software supplied with the instrument (Applied Biosystems). The primers included IL-33 sense, 5’-GTGACGGTGTTGATGGTAAGAT-3’, IL-33 antisense,5’- AGCTCCACAGAGTGTTCCTTG-3’; ST2 sense, 5’- AGAAATCGTGTGTTTGCCTCA-3’,ST2 antisense, 5’-TCCAGTCCTATTGAATGTGGGA-3’; GAPDH sense, 5’-GGAAGGTGAAGGTCGGAGTC-3’, GAPDH antisense, 5’-CGTTCTCAGCCTTGACGGT-3’

### Immunohistochemistry

Formalin-fixed, paraffin-embedded tissues were cut into 4-μm-thick sections, and were dewaxed in xylene, rehydrated and graded ethanol solutions. Antigens were retrieved by heating the tissue sections at 100°C for 30 min in citrate solution (10 mmol/L, pH 6.0). Then sections were cooled and immersed in methanol in the presence of 0.3% hydrogen peroxide for 15 min to block the endogenous peroxidase activity, subsequently rinsed in PBS for 5 min, and then incubated with mouse anti-human IL-33 antibody (0.1μg/ml, Sigma-Aldrich) at 4°C overnight. For negative controls, PBS was used instead of the primary antibody. The sections were then incubated with horseradish peroxidase-labeled goat against mouse/rabbit secondary antibody (Maixin Biotechnology). Diaminobenzene was used as the chromogen and hematoxylin as the nuclear counterstain. Sections were then dehydrated, cleared and mounted.

### Evaluation of staining intensity

All of the staining was evaluated independently by two senior pathologists. Intensity of IL-33 immunochemical staining and the percentage of stained cells in 10 randomly selected fields (×200) were assessed. Each slide was assigned to one of five semi-quantitative groups based on the percentage of stained cells: 0(<5% positive cells), 1(6–25% positive cells), 2(25–50% positive cells), 3(51–75% positive cells), 4(>75% positive cells). The intensity of cytoplasmic and cell nucleus staining was also categorized into four semi-quantitative classes: 0(negative), 1(weak positive), 2(moderate positive), 3(strong positive). Final scores were obtained by sum of the percentage and the intensity scores, and were represented as: -(0–1), +(2–4), ++(5–7). Results from 10 areas were averaged, and then used for the statistical analysis.

### Statistical analysis

Statistical analyses were performed using GraphPad Prism 6.0 software package. One-way ANOVA or Mann-Whitney test was used where appropriate. *P*-values less than 0.05 were considered as being statistically significant.

The Cancer Genome Atlas (TCGA) and Gene Expression Ominibus (GEO) database were used to obtain IL-33 gene expression data in adenocarcimona and squamous cell carcinoma of NSCLC. 5 GEO datasets (GSE30219, GSE37745, GSE42127, GSE50081 and GSE68465) and 1 TCGA dataset were downloaded and reanalyzed in this paper. The expression levels of IL-33 were divided into two groups: high expression group and low expression group. *Cutoff Finder* (http://molpath.charite.de/cutoff) was used to determine a cutoff point. Kaplan-Meier method and the log rank test were used for comparing survival curves.

## Results

### IL-33 was expressed in normal lung tissues

**Fig 1:**
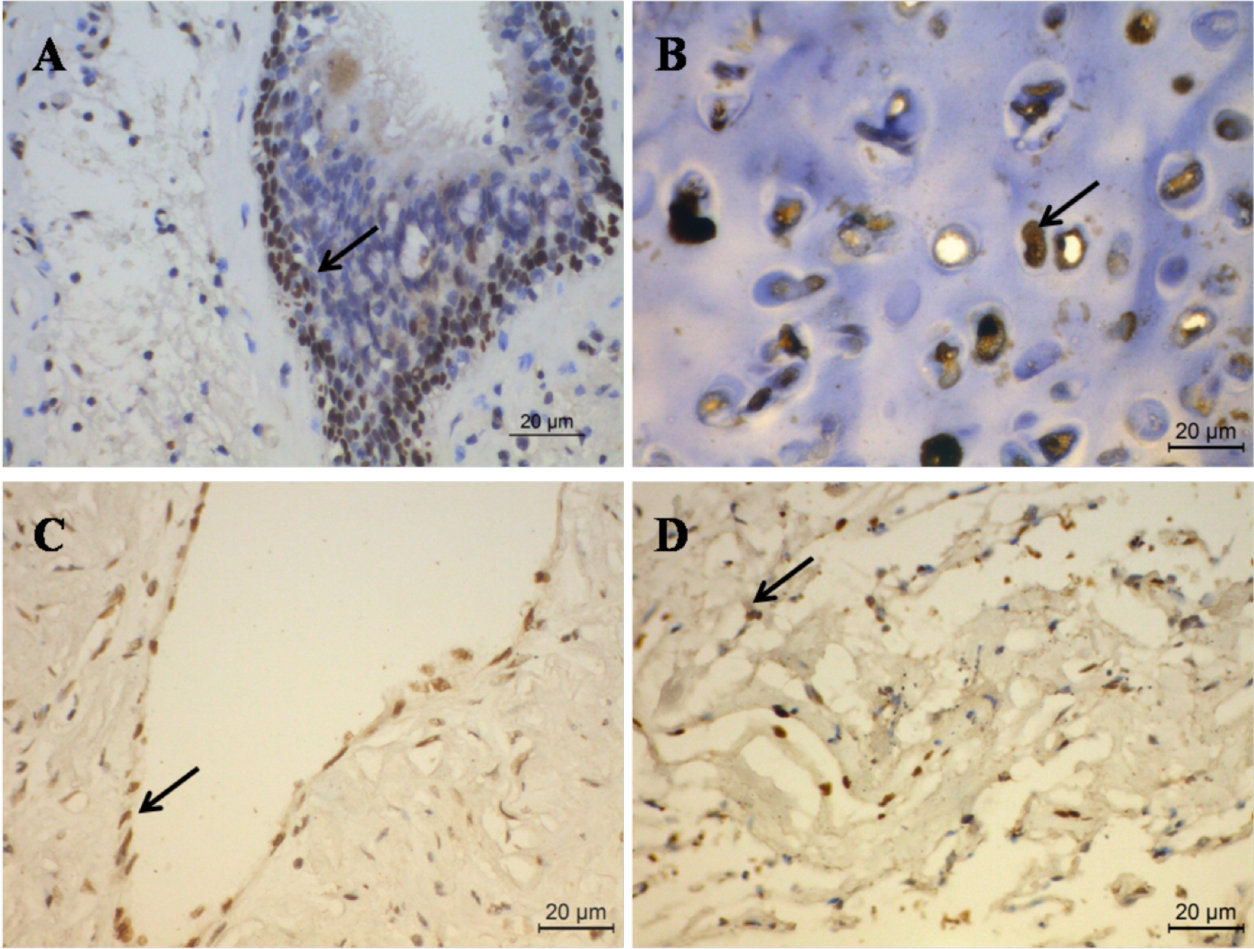
IL-33 is expressed in normal lung tissue. Representative images of immunohistochemistry staining analysis of IL-33 (brown) expression in human lung tissues. (A) bronchial epithelial cells, (B) chondrocytes, (C) vascular endothelial cell (D) alveolus and glandular cells.

We first sought to examine the expression pattern of IL-33 in adjacent normal lung tissues from lung cancer patients. For detection of protein expression of IL-33, we used immunohistochemistry staining. We found that the IL-33 was expressed in various cell types in normal human lung tissues, including bronchial epithelial cells, chondrocytes, vascular endothelial cells and some alveolar and gland cells (Fig 1). Notably, IL-33 was mainly expressed in the nuclei of these cells.

### Expression level of IL-33 was reduced in tumor tissues compared to their adjacent normal tissues in both adenocarcinoma and squamous cell carcinomas

**Fig 2:**
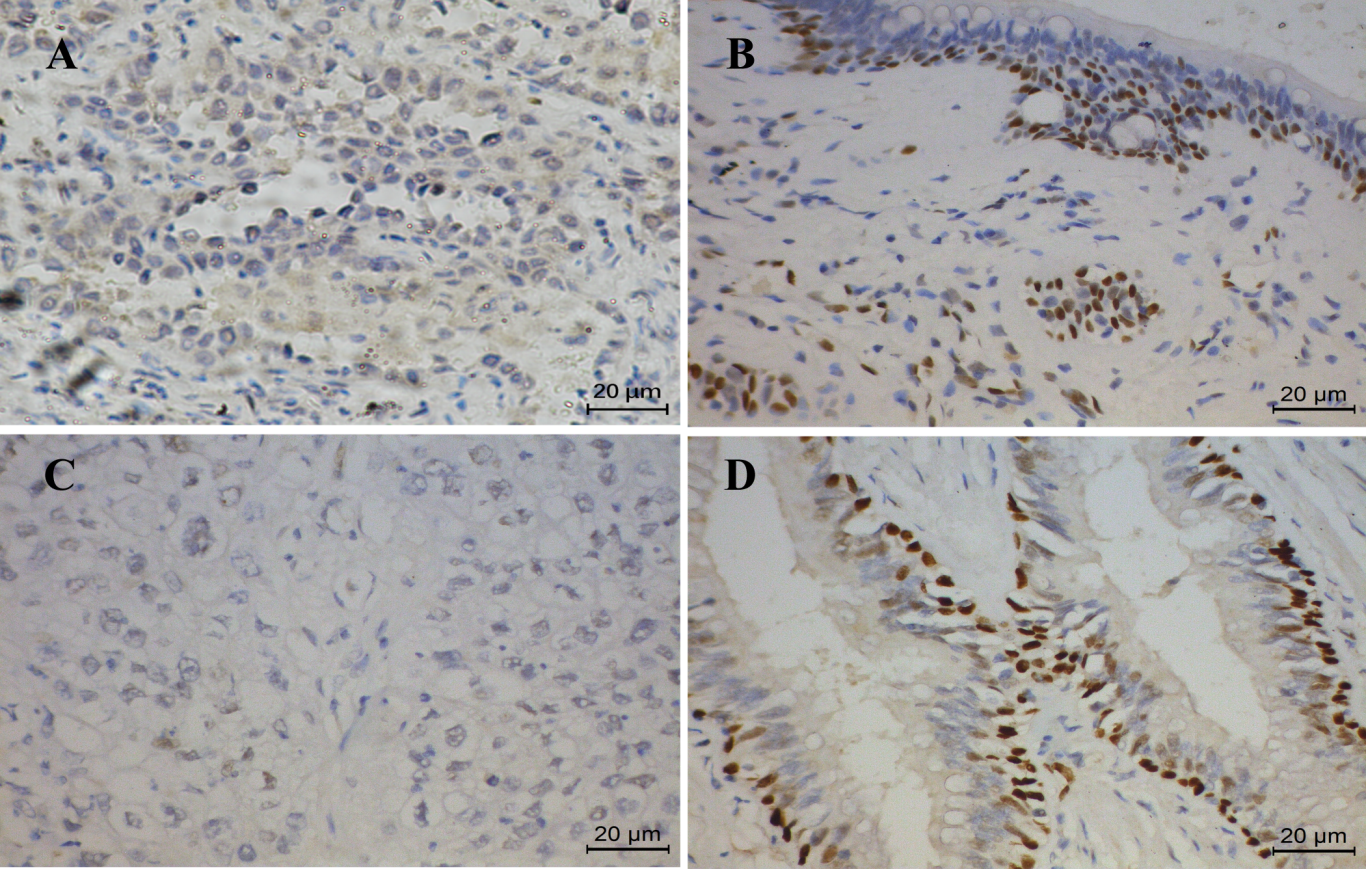
IL-33 is down-regulated in NSCLC. Representative immunohistochemistry staining of IL-33 (brown) in tumor (A, C) and adjacent normal (B, D) tissues from adenocarcinoma (A, B) and squamous cell carcinoma (C, D) in NSCLC.

**Fig 3:**
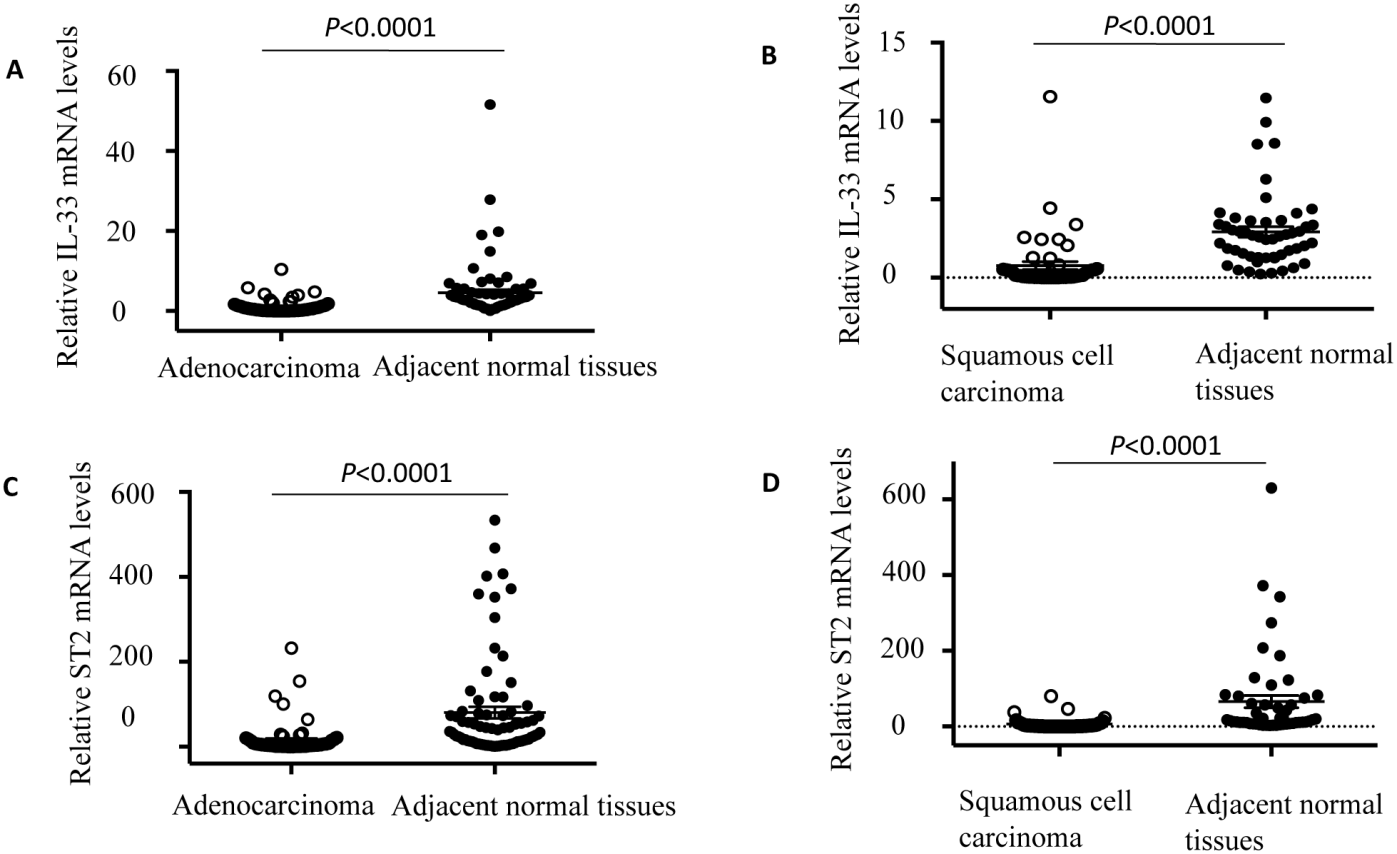
Both IL-33 and ST2 are down-regulated in NSCLC. Statistical analysis of IL-33 and its receptor ST2 mRNA expression levels in adenocarcinoma (A, C) and squamous cell carcinoma (B, D) tissues as well as their adjacent normal tissues from NSCLC patients. n = 80 and 47 respectively, Mann-Whitney test.

Formalin-fixed paraffin-embedded tumor tissues along with their adjacent normal tissues were obtained from 70 NSCLC patients. Among them, 48 patients were diagnosed with adenocarcinoma and the rest 22 were squamous cell carcinoma according to the histological classification. Clinical characteristics were summarized in Tables 1 and 2. We evaluated the protein levels of IL-33 in these samples by the immunohistochemical staining. Our results showed that there were only 27 (56.2%) adenocarcinoma patients with high expression levels (++, intensity score: 5–7) of IL-33 in tumor tissues, while 43 (89.6%) adjacent normal tissues had high expression levels of IL-33 (Fig 2 and Table 3). Squamous cell carcinoma of the lung is distinct from adenocarcinoma in pathological and histological characteristics. For the 22 squamous cell carcinoma tissues we examined, only 5 (22.7%) samples exhibited high IL-33 expression, whereas 20 (90.9%) of the adjacent normal tissues had high levels of IL-33 expression (Fig 2 and Table 4). IL-33 was predominantly localized in the cell nuclei (Fig 2). Consistent with the result of the immunohistochemical staining, when we analyzed the mRNA level of 80 adenocarcinoma samples, we found that the mRNA level of IL-33 was significantly reduced in tumor tissues when compared to their adjacent normal tissues (Fig 3A). Similar results were observed in the other 47 cases of squamous cell carcinoma samples and their matched control normal tissues (Fig 3B). Interestingly, the expression of ST2, a specific receptor of IL-33, was also down-regulated in both adenocarcinoma and squamous cell carcinoma tumor tissues when compared to their matching adjacent normal lung tissues (Fig 3C and 3D). These data suggest that both expression of IL-33 and its receptor were downregulated in lung tumor tissues.

**Table 1:** Expression of IL-33 in adenocarcinomas and its relationship with clinical pathological characteristics of NSCL.

Characteristics	n(%)	IL-33 protein levels			χ^2^ value	P value
		-	+	++		
Sex					2.089	0.3519
Male	22(46)	11	11	0		
Female	26(54)	10	14	2		
Age					4.061	0.1312
<60	18(38)	11	6	1		
≥60	30(62)	10	19	1		
Grading					10.40	0.0342*
Poor differentiated	8(17)	3	5	0		
Moderately differentiated	31(65)	16	15	0		
Well differentiated	9(18)	2	5	2		
Tumor size					0.2233	0.8943
<3cm	28(58)	13	14	1		
≥3cm	20(42)	8	11	1		
N status					1.563	0.4576
N0.1	40(83)	16	22	2		
N2.3	8(17)	5	3	0		
TNM stage					1.957	0.3759
I/IIa	34(71)	13	19	2		
IIb/III/IV	14(29)	8	6	0		

**Table 2:** Expression of IL-33 in squamous cell carcinoma and its relationship with clinical pathological characteristics of NSCLC.

Characteristics	n(%)	IL-33 protein levels			χ2 value	P value
		-	+	++		
Sex						
Male	18(82)	13	5	0		
Female	4(18)	4	0	0		
Age					0.5284	0.4673
<60	6(27)	4	2	0		
≥60	16(73)	13	3	0		
Grading					2.444	0.2946
Poor differentiated	9(41)	8	1	0		
Moderately differentiated	11(50)	7	4	0		
Well differentiated	2(9)	2	0	0		
Tumor size					4.090	0.0431*
<3cm	9(41)	5	4	0		
≥3cm	13(59)	12	1	0		
N status					1.438	0.2305
N0.1	18(82)	13	5	0		
N2.3	4(18)	4	0	0		
TNM stage					0.7487	0.3869
I/IIa	14(64)	10	4	0		
IIb/III/IV	8(36)	7	1	0		

**Table 3:** Summary of IL-33 expression in adenocarcinoma.

	Adenocarcinoma	Adjacent normal tissues	χ^2^ value	*P* value
Low expression IL-33	21(43.8%)	5(10.4%)	13.50	0.0002
High expression IL-33	27(56.2%)	43(89.6%)		

**Table 4:** Summary of IL-33 expression in squamous cell carcinoma.

	Squamous cell carcinoma	Adjacent normal tissues	χ^2^ value	*P* value
Low expression IL-33	17(77.3%)	2(9.1%)	20.84	<0.0001
High expression IL-33	5(22.7%)	20(90.9%)		

### IL-33 expression correlated with clinical and pathological characteristics of lung cancer

To determine the relation between clinical and pathological parameters and the IL-33 expression in adenocarcinoma and squamous cell carcinoma tissues, we then divided the 48 adenocarcinoma and 22 squamous cell carcinoma tissues into 3 subgroups according to the intensity of IL-33 immunohistochemistry staining, i.e., high expression (++, intensity score:5–7), low expression (+, intensity score: 2–4) and negative expression (-, intensity score:0–1) subgroups. Our data showed that lower IL-33 protein expression in adenocarcinoma tissues was significantly associated with higher tumor grade (*P* = 0.0342) (Table 1). IL-33 expression in squamous cell carcinomas inversely correlated with tumor size rather than tumor grade (*P* = 0.043) (Table 2). These data demonstrate an inverse correlation between IL-33 expression and lung cancer progression.

### Higher IL-33 expression in tumor was associated with longer survival of NSCLC patients with adenocarcinoma

**Fig 4:**
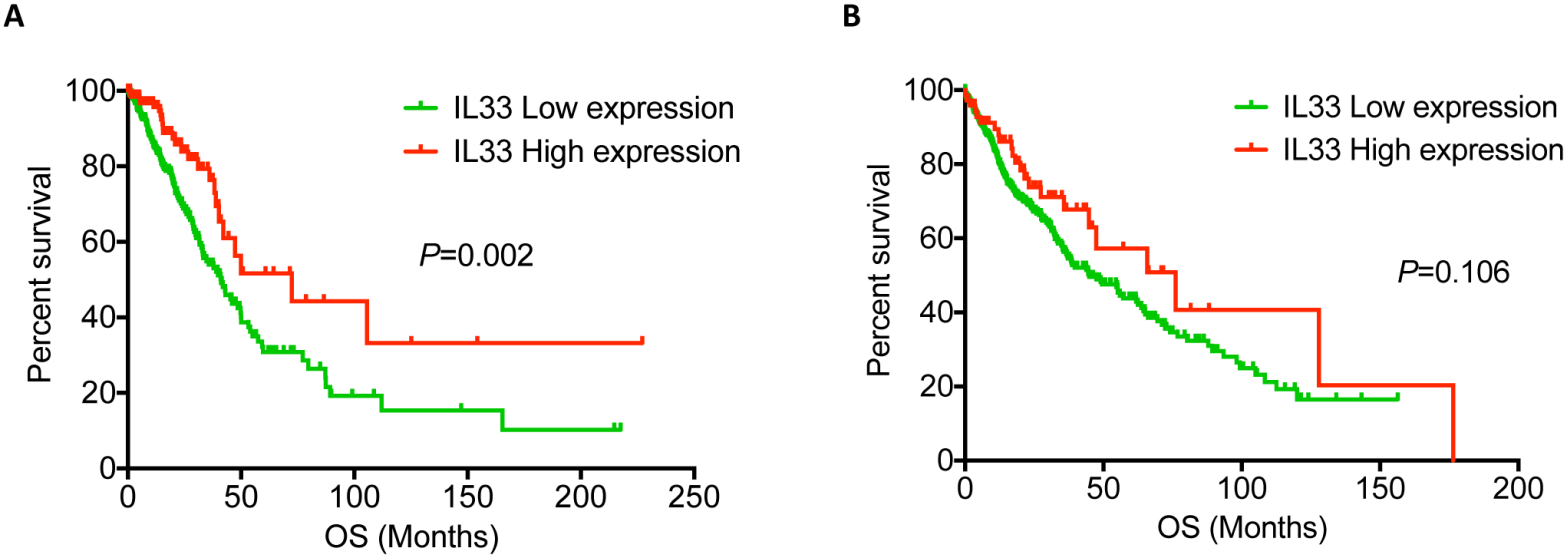
Higher levels of IL-33 mRNA were correlated with prolonged overall survival of adenocarcinoma NSCLC. Survival analysis of Adenocarcinoma (A) and squamous cell carcinoma (B) with high or low IL-33 expression in NSCLC patients. Data are collected from TCGA. Log-rank test was performed.

We have shown that IL-33 was down-regulated in tumor tissues compared to adjacent normal tissues and its expression in tumor was inversely correlated with tumor grade in adenocarcinoma and with tumor size in squamous cell carcinoma of NSCLC. We then decided to determine whether IL-33 RNA expression level was correlated with overall survival of NSCLC patients. To this end, we downloaded and reanalyzed five independent lung cancer datasets from The Cancer Genome Atlas (TCGA) and the Gene Expression Ominibus (GEO) database. We examined the relationship between IL-33 expression in tumor tissues and the overall survival time in patients suffering from adenocarcinoma of the lung. Upon analyzing the TCGA dataset, we found that patients with higher expression levels of IL-33 RNA had longer overall survival time (Fig 4A). Strikingly, we reached the same conclusion with four other independent datasets from the GEO database (S1A–S1D Fig). In contrast, association between higher IL-33 expression and survival was less consistent for squamous cell carcinoma of the lung among different datasets. (Fig 4B and S1E–S1H Fig). We also performed analysis on ST2 and could not find any correlation between ST2 levels and survival (S2 Fig). These data indicated that higher expression levels of IL-33 RNA were associated with better survival of lung adenocarcinoma but not lung squamous cell carcinoma.

## Discussion

In this study, we showed that IL-33 as well as its receptor ST2 were significantly down-regulated in both adenocarcinoma and squamous cell carcinoma of the lung when compared to adjacent normal lung tissues. And the level of IL-33 protein was inversely correlated with tumor grade and size. Analysis of five independent TCGA and GEO lung cancer expression datasets further revealed that higher expression levels of IL-33 mRNA were associated with longer overall survival of adenocarcinoma patients. These data are consistent with the hypothesis that IL-33 has an antitumor role in NSCLC.

We demonstrated that IL-33 was expressed in alveolar type II cells in normal human lung tissues. In addition, after examination of data from the protein atlas database, we also found that IL-33 expression could be readily detected in the nuclei of type II alveolar cells in normal human lung. In contrast, IL-33 expression in lung tumor cells was much reduced. We believe this is true down-regulation of IL-33 during tumor progression because, based on mouse studies, alveolar type II cells are believed to be the cell origin of lung adenocarcinoma. For example, tumors and premalignant lesions in both Kras (the most common mutation for adenocarcinoma) and Kras/p53FL/FL models have been shown to be positive for both Sp-B and Sp-C, attesting to the alveolar type II cell origin of the tumors [15,16]. Many recent mouse studies further confirmed that the alveolar type II cell as the cellular origin of lung adenocarcinoma [17–19]. Since IL-33 was highly expressed in normal alveolar type II cells in both human and mice and expression was much reduced in lung adenocarcinoma cells, we believe it is appropriate to conclude that IL-33 is down-regulated in adenocarcinoma of lung compared to normal tissue cells. Tracheal basal cell progenitors are thought to be the cell origin of mouse lung squamous cell carcinoma (SCC) [20,21]. We showed that IL-33 was expressed by basal cells of trachea and down-regulated in human lung SCC. Therefore, it is also appropriate to conclude that IL-33 is down-regulated in SCC of lung compared to normal tissue cells. Lastly, TCGA data are usually well quality-controlled by the amounts of tumor areas of the dissected tissues and in general similar amounts of tumor tissues were included in RNA preparation. Therefore, the RNA level should reflect the true levels of tumoral expression of IL33 rather than the amount of normal tissue contamination. Collectively, these data support the idea that IL-33 down-regulation in lung epithelial cells is associated with malignant transformation.

Large bodies of evidence indicate that IL-33 has a potent anti-tumor function in many cancers. In a study of mouse B16 melanoma and Lewis lung carcinoma models, IL-33 was shown to inhibit metastasis [22]. In our previous study, we found that tumoral expression of IL-33 greatly inhibited tumor growth and metastasis [23]. Mechanistically, ST2, a well-defined receptor of IL-33, is expressed on CD8^+^ T cells, Th1 cells, NK and NKT cells [9,10] and IFN-γ production by tumoral CD8^+^ T cells and NK cells was significantly increased by IL-33 [23]. Furthermore, the antitumor effect of IL-33 is dependent on CD8^+^ and NK cells [23]. Therapeutically delivery of IL-33 protein can inhibit the growth of established tumor by inducing robust anti-tumor immunity of CD8^+^ T cells [24]. In addition, IL-33 can restore DC activation and maturation by interacting with its receptor ST2 on DC to induce co-stimulatory molecule expression cells [24]. In these settings, IL-33 triggers the type I antitumor immune responses by having a direct effect on CD8^+^ T cells, NK cells and DC. Consistent with these mouse data, IL-33 has been shown to be reduced in many epithelial cancer cells including breast cancer, colorectal cancer and cervical cancer. [25,26]. Analysis of the clinical data shows that expression levels of IL-33 are reduced during tumor progression in breast cancer [26] and inversely correlate with tumor grade [27]. These data indicate that downregulation of epithelial IL-33 is associated with tumorigenesis in breast cancer. In addition, ST2 is also shown to be reduced in human colon cancer and inversely correlated with the tumor grade [28]. Consistent with the antitumor role of IL-33, IL33-deficient mice are highly susceptible to colitis and colitis associated cancer [29]. Collectively, these data support an antitumor role of IL-33.

IL-33, however, has also been suggested to play a protumor role in some experimental settings. Overexpression of IL-33 in human lung tumor cells promotes their growth in immune deficient mice [30,31]. Breast cancer cell line 4T1 showed decreased metastasis in ST2-/- mice [32]. This is attributed to the direct effect of IL-33 on myeloid derived suppressor cell (MDSC) [12]. In a mouse colon cancer model using the MC38 cell line, it is shown that IL-33 level is significantly elevated in colorectal cancer liver metastasis and overexpression of the full-length IL-33 can promote tumor growth and increase liver metastasis incidence through myeloid cell recruitment [33]. And another study shows that overexpression of mouse IL-33 promotes colon cancer cell “stemness” and growth *in vivo* [34]. In this model, expression of the full-length IL-33 was rendered by the CMV promoter, a relatively weak promoter, resulting in the release of a small amount of extracellular IL-33. The minute amount of extracellular IL-33 promotes tumor growth through recruiting MDSC but cannot have any significant effect on type 1 lymphocytes, likely due to differential expression of ST2 on MDSC and CD8^+^ T cells. It is also possible that the full-length IL-33, due to its primary localization to the cell nucleus, has a unique protumor function [25].

Mast cells, M2, and Treg are also potential mediators of IL-33’s protumor function. A modest reduction of the number and size of polyps in small and large intestines were observed in IL-33^-/-^ APC Min/+ mice compared to WT APC Min/+ mice. Decreased polyposis is associated with decreased mast cells which are crucial for polyps formation in this model [35] [36–38]. It is quite likely that, in the APC Min/+ model of polyposis, IL-33 promotes polyposis formation via Th2 cytokines IL-4 and IL-13 as well as inflammatory cytokine IL-6, which facilitate mast cell function. In the same vein, another study shows that ST2 deficiency does not affect polyposis in large intestine but does reduce polyposis in small intestine in the APC Min/+ model. This study further shows that IL-33 promotes Treg and M2 in the APC Min/+ model. IL-33 primarily targets Treg, mast cells and M2 rather than CD8^+^ T cells in the APC Min/+ model, because APC mutation results in activation of the WNT pathway, which inhibits CD8^+^ T cell recruitment [39].

In summary, the effect of IL-33 on tumorigenesis is dependent on its predominant target cell types. High levels of IL-33 in the tumor tissue can drive anti-tumor responses via CD8^+^ T cells, NK cells, NKT cells and DC. Whereas low levels of IL-33, combined with a dampened type 1 lymphocyte-mediated antitumor immune response, promotes tumor progression through Treg, MDSC, M2 and mast cells. Thus, IL-33 can promote type 1 immunity against cancer in the setting where a cancer therapy induces tumor cell death and release of large amounts of IL-33. This might explain why lung cancer patients with higher expression levels of IL-33 survive longer. Nevertheless, the exact role of IL-33 in lung cancer development needs to be established using appropriate mouse models of lung cancer.

## Supporting information

S1 Fig Survival analysis of adenocarcinoma (A-D) and squamous cell carcinoma (E-H) with high or low IL-33 expression in NSCLC patients. Data are collected from GEO database. Accession numbers are (A, F) GSE37745 (B, H) GSE42127 (C, G) GSE50081 (D)GSE68465 (E)GSE30219. Log-rank test was performed.
(PPTX)

S2 Fig Survival analysis of adenocarcinoma (A) and squamous cell carcinoma (B) with high or low ST2 expression in NSCLC patients. Data are collected from TCGA. Log-rank test was performed.
(PPTX)
