# Supplementary figures and images for: Lower expression level of IL-33 is associated with poor prognosis of pulmonary adenocarcinoma
Source: PLoS One. 2018 Mar 2;13(3):e0193428. doi: 10.1371/journal.pone.0193428 (PMC5834175; doi:10.1371/journal.pone.0193428)

## Slide 1
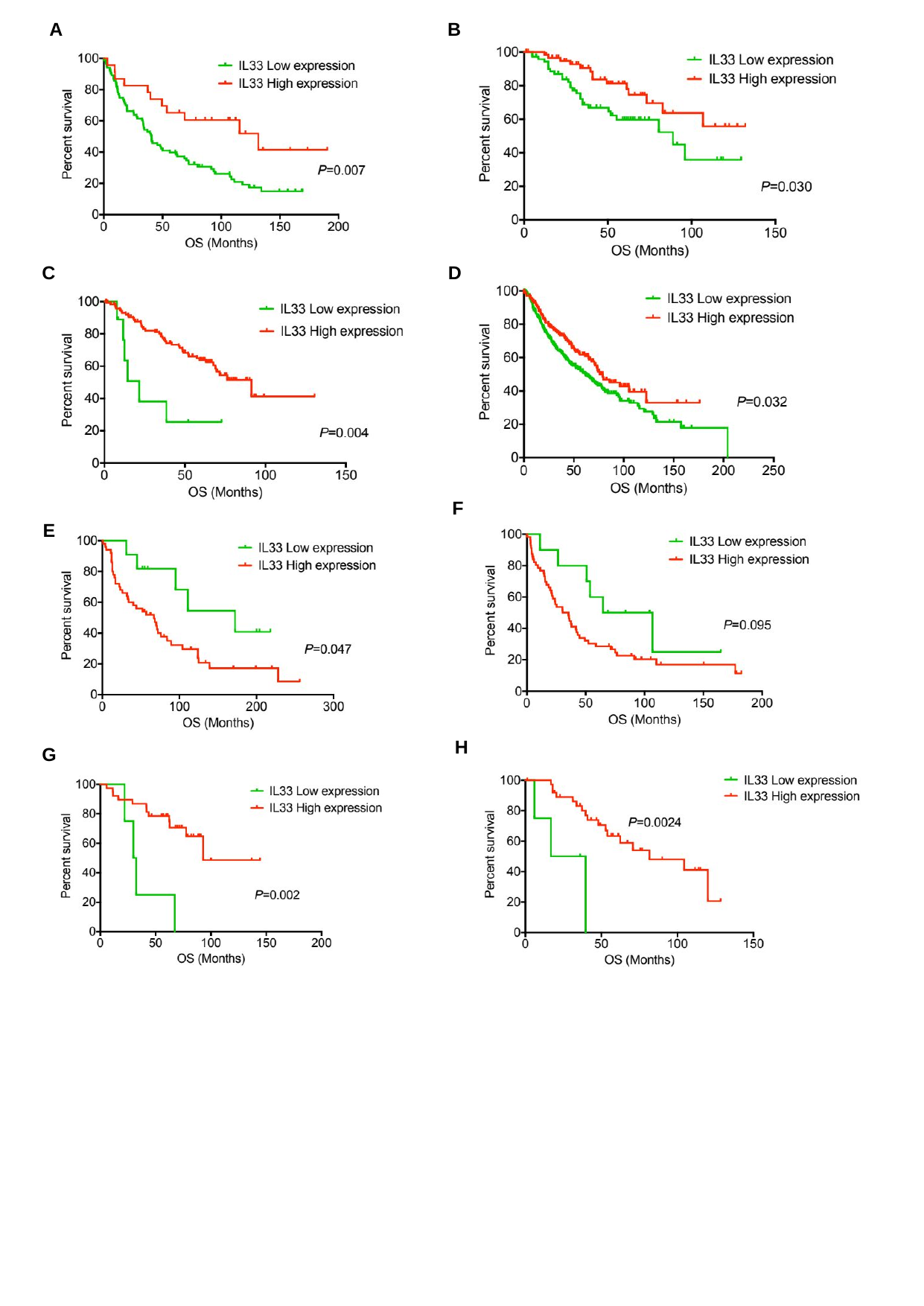

A
B
C
D
F
E
H
G

Supplement: S1 Fig — Data are collected from GEO database. Accession numbers are (A, F) GSE37745 (B, H) GSE42127 (C, G) GSE50081 (D)GSE68465 (E)GSE30219. Log-rank test was performed. (PPTX) [file pone.0193428.s001.pptx]

## Slide 1
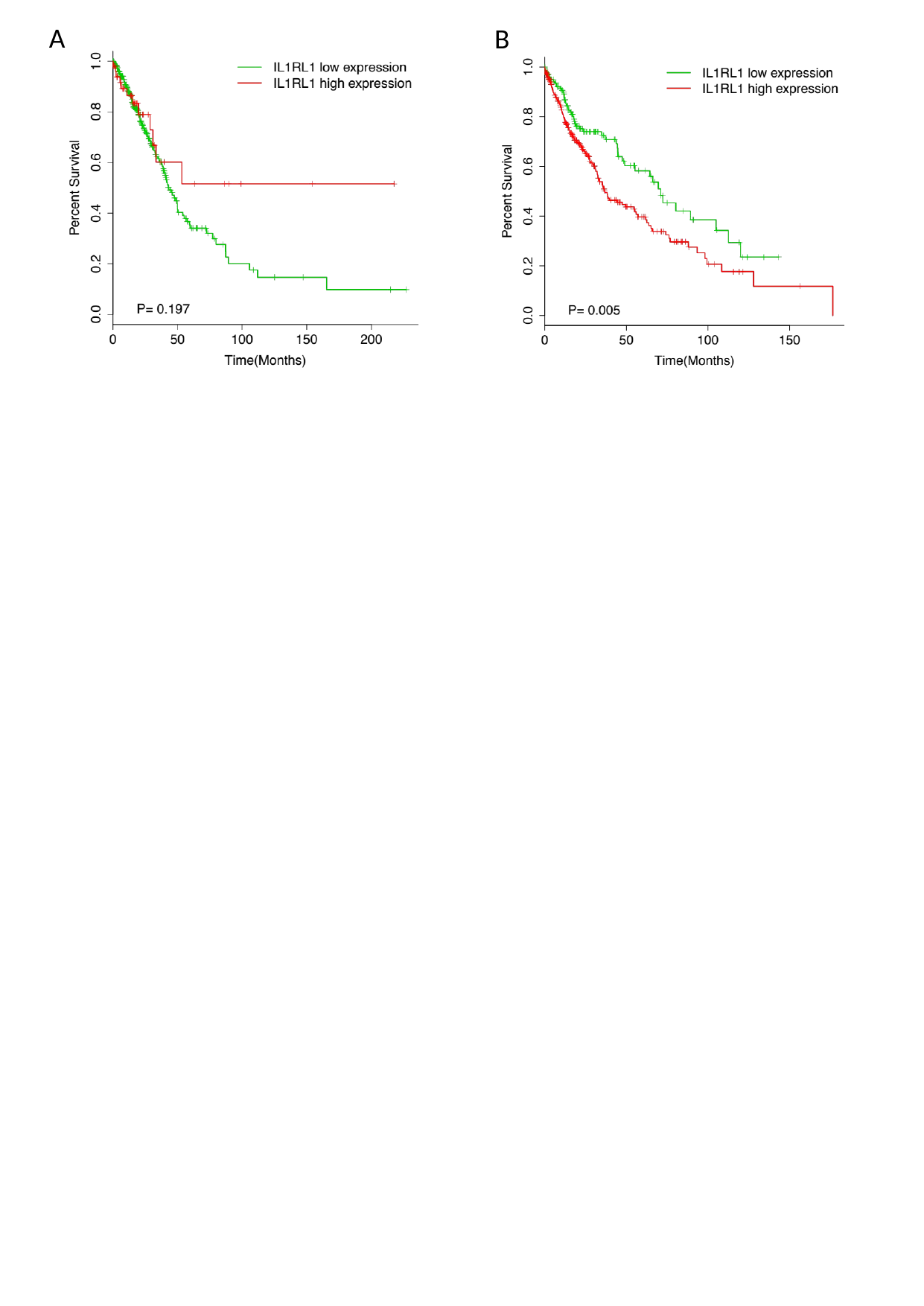

A
B

Supplement: S2 Fig — Data are collected from TCGA. Log-rank test was performed. (PPTX) [file pone.0193428.s002.pptx]
